# Exploring Women’s Multi-Level Career Prospects in Pakistan: Barriers, Interventions, and Outcomes

**DOI:** 10.3389/fpsyg.2019.01376

**Published:** 2019-06-19

**Authors:** Ambreen Sarwar, Muhammad Kashif Imran

**Affiliations:** ^1^Department of Management Science, Comsats University Islamabad, Lahore, Pakistan; ^2^Department of Management Science, The Islamia University of Bahawalpur, Bahawalpur, Pakistan

**Keywords:** relational framework, diversity management, multi-level analysis, Pakistan, workplace gender parity, working women, Nvivo

## Abstract

Utilizing the relational framework of diversity management and social role theory, this exploratory study illuminates the career prospects of women working in the patriarchal society of Pakistan. With the help of Nvivo 12 Plus, themes were generated based on 27 in depth, semi-structured interviews. The findings showed an interrelated web of factors interacting at three levels; macro, meso, and micro; that were explained on the premises of the social role theory. Major impediments to women’s career progress were religious interpretations, socio-cultural factors and Sifarish (i.e., favoritism/nepotism) at the macro-level. At the meso-level, the barriers involved segregation, discrimination, harassment, and lack of work-family balance initiatives at the workplace. The micro-level obstacles involved personal values and needs, queen bee syndrome and a lack of awareness. The experiences of Pakistani women varied based on socio-economic status, marriage, religion and various aspects of individual identity, that intersected with gender and accordingly affected their career outcomes either positively or negatively. Therefore, the study also contributes to the emergent area of intersectional studies which posits that individuals experience various facets of identity concurrently and that these factors do not operate separately but are interrelated and interact. Moreover, the study also highlights the interventions for creating gender parity like clarification of Islamic guidelines, strict accountability of workplace policies, and the provision of work-family balance support. The outcomes highlighted by the respondents included happiness, confidence, agency, and improved quality of life. The paper concludes with implications for theory and practice, limitations, and future research avenues.

## Introduction

“Women’s career inequality harms not only women but families and society”

                                                                                                                                    (Kossek et al., 2016, p. 249)

Extant literature (Joshi et al., 2015a,b; Ghouse et al., 2017; Madsen and Scribner, 2017; Van Oosten et al., 2017) signals an up-surging trend in studies related to workforce gender diversity since it is on an increase globally (Ali et al., 2011). Women are entering the workforce in greater numbers (Kiaye and Singh, 2013; Ng and Sears, 2017) and are also represented at top positions in government, business, and education (Adler, 2015). This upsurge is relevant for factors like diversity programs, mentoring for women (Madsen and Scribner, 2017), equity programs (Jain et al., 2010; Kiaye and Singh, 2013), progressive work life balance policies (Adame et al., 2016), and rising education levels (Turcotte, 2011) among others. According to the United Nations: “Gender equality is at the very heart of human rights” (Women’s Human rights and gender equality, 2017). Therefore, impartial access to education and employment has been acknowledged as not merely a women’s concern but as a human rights issue (UN Women, 2016). Numerous women belonging to some developed and developing nations are increasingly obtaining university degrees (Klasen and Pieters, 2015). Nevertheless, increasing education and policy interventions fail to translate into a proportionate increase in representation of women in the work force in various regions of the world; making the progress toward equitable workplace promising, yet intricate.

According to the McKinsey Global Institute (2017), women represent a large part of untapped labor. As compared to men, 655 million fewer women are economically active worldwide. Though, amongst higher-education graduates, 50% are women, yet at management positions they appear to represent only 25%. Additionally, they are accountable for 75% of unpaid care work (McKinsey Global Institute, 2017). In light of the social role theory (Eagly and Steffen, 1986), such an imbalanced division of paid and care work might be a result of variances in role occupancy in different cultures, households, and professions which creates role expectations for each gender.

“In many countries, women have a higher level of education than men but earn lower wages, even when they work in the same professional fields”

                                                                                                                                    (Vázquez-Álvarez; as cited by Varela, 2018)

Impediments to female’s career advancement and discrimination are more pronounced in developing and under developed nations. If nations and organizations are willing to tap this potential workforce to further their economic and communal cause, a better understanding of the lived experiences of women personnel is required in developing countries. Madsen and Scribner (2017) reported on a shortage of research related to gender in several areas of the world. Out of the 152 articles they analyzed, developing nations like Pakistan (and others) were represented in only one study. Other scholars also emphasize the need to progress their discipline by taking on a less Western-centric perspective (Holden et al., 2015; Madsen and Scribner, 2017; Ng and Sears, 2017). Additionally, according to Syed and Ali (2013) there is an increasing necessity to illuminate the career related experiences of females in key Islamic nations. Therefore, the purpose of this qualitative study is to explore the career prospects and experiences of working women employed in various industries of an Islamic country; Pakistan. Drawing on in depth, semi-structured interviews with 27 working women within the workplace context of Pakistan, this study addresses the multi-level barriers to gender equality, multi-level interventions that might boost gender equality, and multi-level outcomes of gender equality.

Though women’s work force participation in Pakistan (13.4% in 1990; 24.5% in 2016), a major Islamic state, has increased alongside gross domestic product (GDP), it remains well-below the levels in other countries with similar incomes (World Bank, 2016). Pakistan stands at second last place (143^rd^ out of 144 countries) in the World Economic Forum’s Global Gender Gap index with respect to gender equality (GGGR, 2017) just one rank ahead of war-torn Yemen (ranked at 144^th^). Another recent report from the International Labor Organization has mentioned Pakistan as worst in terms of the gender pay gap (Varela, 2018). According to Pio and Syed (2013) almost four out of every five Pakistani adult females do not represent the work force. The situation for educated and qualified women is not so different, since only about 25% of Pakistani women, who have a university degree, work outside the home (Tanaka and Muzones, 2016). Such a shortage of women in the labor force results in a significant potential loss of productivity in economic growth. It is now time that we start identifying and eliminating the root cause of female career inequality and begin to focus our attention on an all-inclusive analysis of the issue in Pakistan.

Earlier studies carried out in Pakistan includes research related to equal employment opportunities (Ali, 2013), modesty and honor (Rehman and Azam Roomi, 2012; Syed and Ali, 2013), and the legislative framework of Pakistan (Mullally, 1995; Goheer, 2003; Özbilgin et al., 2012). However, it has been observed that important concepts, such as the dual-centric careerist orientation of women professionals, queen bee syndrome and implications of Sifarish (i.e., favoritism/nepotism) for working women has been neglected. Furthermore, some researchers studying women’s employment in Pakistan (e.g., Jejeebhoy and Sathar, 2001; Fakhr et al., 2016), have emphasized national or organizational level factors exclusively (e.g., Rehman and Azam Roomi, 2012; Sarwar and Abbasi, 2013), and have failed to pay attention to the problem, its solution and outcomes holistically. We believe that for addressing this gap and for a more comprehensive understanding of the intricate interaction of factors influencing and that are influenced by gender equality, the relational framework of diversity management (Syed and Özbilgin, 2009) be utilized, which integrates three interrelated levels: macro-societal, meso-organizational, and micro-individual.

We contribute to the extant literature by identifying and incorporating the various levels at which gender inequality interact. We illuminate the understanding of major barriers women come across during their career course, along with the outcomes of and interventions that organizations can employ to improve women’s career experiences; thereby answering calls of Joshi et al. (2015a) and Madsen and Scribner (2017) who ask us “to challenge ourselves as engaged scholars to go both broader and deeper into understanding the many complexities” (p. 1147) regarding gender issues on the global stage, as well as to the call of others who suggest studying holistic multilevel gender diversity related issues (Zanoni et al., 2010; Sarwar and Abbasi, 2013; Theodorakopoulos and Budhwar, 2015). While doing so we also utilize the theoretical lens of ‘social role theory’ to describe the work-related experiences of working women in Pakistan.

The key questions that this research concentrates on are:

• What are the multi-level barriers to gender equality in Pakistan?
• What are the multi-level interventions that might boost gender equality in Pakistan?
• What are the multi-level outcomes of gender equality in Pakistan?

### Workplace Gender Parity: A Multi-Level Phenomenon

Workplace gender parity is a multilevel, multidisciplinary intricate phenomenon. It signals the extent to which women, in comparison to men (a) enjoy equivalent access to and participation in professional prospects and (b) encounter identical work and non-work consequences (Kossek et al., 2016, p. 229). Such parity can bear fruitful outcomes not only for organizations, but for the nation, society and women at large. Syed and Özbilgin (2009) theorized a multi-level approach to gender diversity that links three levels of analysis, i.e., the macro-societal, meso-organizational, and micro-individual. Such a linking mechanism is helpful to arrive at a more inclusive, rational, and context-specific framing of diversity management. We believe that this approach is effective for a complete understanding of individual choices, organizational processes and structural conditions, in the presence of national and societal characteristics which collectively explain the persistent power disparity and career disadvantage for female professionals within a social and employment settings. At the macro-social level, the relational framework considers the consequence of national constructs and establishments like laws, institutions, culture, political economy (Syed and Özbilgin, 2009) and religion. At the meso-level, the organizational procedures and behaviors at the workplace are studied. Finally, the micro-individual level comprises of individual’s personal choices, strengths, and weaknesses.

The behaviors thought to exist in a social context, are consequently expected to occur in organizations, leading to the way we act (Scott, 2014). Scott’s (2014) assertions proposed that people in organizations choose to act in accordance with the entrenched societal principles that construct the communal interface (Hodgson, 2006, p. 13), in addition to official rules. With respect to gender parity at the workplace, social structures based on traditions and other norms might be anticipated to have a lasting impression (Cain et al., 1979). Institutions are intricate, enduring social arrangements that display a great amount of strength and offer assistance, sense, and steadiness to communal life (Scott, 2014). Institutions utilize customs and regulations as guiding principles for communal actions and encompass cultural-cognitive, normative, and regulatory pillars (Scott, 2014). Individuals live and work within such contexts and affect as well as are affected by such norms and rules from an early age. Due to such social contextual effects, processes and outcomes are interconnected at national, organizational, and individual levels. With better gender diversity across an organization, women gain personally (Joshi et al., 2015a,b), as well as firms (Krishnan and Park, 2005) and society (Kossek et al., 2016). Gender parity in the workplace involves connecting ideas like career, family, and gender predisposition (Martins et al., 2002). Considering these arguments, workplace gender equality would more likely occur over a life and career course with interrelated work and non-work connections.

#### Macro-Level

At the macro level, issues like expected social roles (Weyer, 2007; Loots and Walker, 2015; Al-Asfour et al., 2017), cultural values (Ali, 2013; Ghouse et al., 2017; Syed et al., 2018), patriarchal ideology, religion (Ali, 2013; Pio and Syed, 2013; Syed and Ali, 2013; Al-Asfour et al., 2017; Syed et al., 2018), legislative and national level policies (Ghouse et al., 2017; Nielsen, 2017b; Syed et al., 2018) are significant to consider. These issues impact the stiffness of customs for gender role correspondence and socialization (Eagly, 2013) which shape gender dissimilarities in actions that are embedded in social norms regarding capability and anticipated behaviors/roles of men and women at the workplace and in society (Kossek et al., 2016). Hofstede (2001) claims that gender roles are stiffer in masculine cultures, where men are anticipated to govern society. The patriarchal, collectivist, and masculine nature of societies poses harmful effects on the careers of women, creating several challenges and impediments to employed women (Al-Asfour et al., 2017). National norms encouraging traditional patriarchy do not support women to go out of the home for a job or to obtain assistance for taking care of children. Metcalfe (2007) reflects on how women in conservative societies are anticipated to quit their professional careers after marriage and are expected to refrain from interacting with men.

Syed et al. (2005) studied the influence of the religious-cultural environment on communal anticipations of feminine modesty. For example, in Islamic customs the husband is held accountable to financially bear the expenses of the spouse and offspring; consequently, there is comparatively restricted encouragement and opportunity for women to participate in remunerated employment. Although Islamic commandment allows female employment (Al-Asfour et al., 2017), the actual outlook of Islamic settings for working women and their conduct at a job are rather tougher as compared to those for men (Syed et al., 2005). Regulatory and legal backgrounds also affects gender parity. Beller (1982) noticed that female employees in the United States benefited in the 1970s when several legislative acts and orders made affirmative action a mandatory function of national contractors. In the same vein, research concentrating on Chinese women’s careers disclosed that the attainment of better female participation in paid work owes much to the endeavors of the state (Cooke, 2001). Such results signify the importance of endeavors for equal employment opportunity laws that foster organizational changes, which lessen the undesirable influence of gender stereotyping. These arguments stress the need for ongoing legal pressures to warrant the sustained female advancement toward ranks of influence and authority. Gender disparity is therefore rooted in this network of religious opinions, communal customs, norms, and religious and legislative guidelines.

As a policy intervention, various scholars (e.g., Beller, 1982; Leonard, 1986; Blau and Beller, 1988; Jacobs, 1992; Kelly and Dobbin, 1999; Terjesen et al., 2015) have put forward national level institutional bindings as vital factors in administrative structures and a leading influence in improving the position of underprivileged groups, like women. Lately, Pakistani governments have shown a significant level of determination to improve women’s career experience by adopting various policy interventions like ratifying key international conventions, legal policies, local labor laws, labor protection policies, equal pay and employment opportunities (for details see Ali, 2013). However, recent numbers (GGGR, 2017) display a worrying disparity between policy and practice in Pakistan. Our study highlights why, despite stipulating progressive policy interventions, gender disparity still prevails in the country and what additional steps might be taken to further down the gender gap. The literature shows gender equality might bear fruitful outcomes at the macro-level including improvement in GDP (Aguirre et al., 2012), increased labor productivity (Scott-Jackson et al., 2010) and reducing poverty in developing nations (Elder and Smith, 2010).

##### Social role theory at the macro-level

In light of the social role theory, men and women are disseminated with differing social roles due to human’s evolved bodily gender variances where men are bigger, quicker, and have more strength, whereas women give birth and nurture offspring (Eagly and Wood, 2011). Similarly, the Quranic teaching by some scholars are interpreted as allocating gender roles by portraying men as breadwinners of the family and women as caregivers (Khalaf, 2017). It might be the foremost reason behind the discrimination between genders, when it comes to paid work. Such a division of gender roles has been manipulated and used by some extremists to proclaim their domination over women, interpreting the Quranic guidelines in accordance with their own patriarchal comforts resulting in a society more prone to gender discrimination.

Additionally, the social role theory (Eagly and Steffen, 1986) posits that men are anticipated to have agentic traits, while women are expected to show communal traits (Diekman and Eagly, 2008), therefore women may more regularly have to face circumstances and condemnations that they are deficient in hardiness, and hence face more problems in the construction of facilitating relations and networks (Timberlake, 2005). The gender segregation produced due to the social roles prevailing in patriarchal societies like Pakistan, reproduces career inequality by providing women limited access to power, opportunities, and networks, as compared to men (Kanter, 1977) and therefore the issue of Sifarish becomes more problematic in the conservative culture of Pakistan which is largely adopted from the Hindus of the Indo-Pak subcontinent and not from the Islamic guidelines of merit. Therefore, the social roles prevailing at the macro-level generates a religious-cultural environment of communal anticipations of male dominance and supremacy resulting in a lack of gender parity in the whole society.

#### Meso-Level

The meso-organizational level concentrates on organizational processes, behaviors, routines, and outcomes that influence workplace gender equality. These factors affect and are affected by the macro-level society and micro-individual level employees. It is interesting to observe that at this level, gender inequality is noticeable not only in the form of consequences like decreased female workforce participation but also in processes, e.g., gender prejudice in selection and advancement (Alimo-Metcalfe, 2010), gender segregation (Acker, 2009), harassment (Berdahl and Moore, 2006; Ali, 2013) exclusion of females from male progressive networks (Timberlake, 2005; Abalkhail and Allan, 2015), the inadequate family supportive strategies like maternity leave and structural facilities like child care services, and coercing female employees out of the labor force after having babies (Metcalfe, 2007; Al-Asfour et al., 2017). It is vital to emphasize the interconnectedness of the macro-, meso-, and micro-level variables as the impeding factors of gender parity which cannot be comprehended by viewing these barriers distinctly. For instance, nation’s labor market is mainly characterized by the socio-economic and normative factors. Women’s career options in the market are inadequate and restricted by the cultural constraints of “female modesty” or “purdah” (Ali, 2013).

Secondly, even though governmental and organizational policies exist to improve female employment (Ali and Knox, 2008), e.g., the laws against harassment, most female employees hesitate to report such incidences (Ali, 2013) for the fear of being marked as immodest. This reflects a personal choice at the micro-level but is influenced at the same time by the macro-level contextual factors that impart a female child from the beginning, the concepts of gender segregation (Balchin, 1996; Ali, 2013; Al-Asfour et al., 2017). Furthermore, discrimination in the work-related practices prevail, for example in recruitment, selection, training, career advancement, and equal wages and rewards usually fail to diagnose the macro-level impacts that in turn effect gender discrimination at the meso-organizational level (Al-Asfour and Khan, 2014). Male employees and leaders are usually overwhelmed by socio-cultural factors from the larger context of society, and apply those at workplaces, resulting in workplace gender inequality.

Earlier researchers (e.g., Weyer, 2007; Broadbridge, 2008; Brough and O’Driscoll, 2010; Tlaiss and Kauser, 2010; Ali, 2013; Nielsen, 2016; Al-Asfour et al., 2017) have proposed several meso-level interventions that might render helpful in inducing gender parity in organizations, like a flexible work environment, merit based HR practices, incentives based programs, enforcement of harassment and equal opportunity employment laws, work-life balance programs, and mentoring and networking programs. Our study takes another step forward by giving a voice to the Pakistani working women in terms of what interventions are being adopted by their respective organizations and what further steps might be adopted to improve their work-related experiences. The extant literature highlights various positive outcomes of such initiatives (Krishnan and Park, 2005; Smith et al., 2006; Francoeur et al., 2008).

##### Social role theory at meso-level

In light of the social role theory (Eagly and Steffen, 1986), since the gender roles of men and women are entrenched in traditional principles regarding the capabilities and anticipated actions of men and women in paid and household spheres, they also define the extent to which management practices are gendered in execution (Kossek et al., 2016) at the meso-level and results in gender bias, and segregation at the workplace in patriarchal cultures like in Pakistan. Similarly, as the social role theory posits that women are expected to be more communal, it is a possible reason that in the conservative culture of Pakistan, they are harassed and stereotyped more because of the assumption that they lack hardiness. Women, as an outcome of prearranged gender roles, might come across as two types of preconceptions in work settings (Eagly and Karau, 2002); the descriptive prejudice and the prescriptive prejudice. The former is based on the stimulation of descriptive beliefs about women’s stereotypical attributes, that are usually not the required and desired qualities of leaders (Diekman and Eagly, 2008). Therefore, women might not be positively evaluated for potential paid work, management and leadership positions creating an environment of inequality at the meso-organizational level. The later has its roots in preconceptions about appropriate female behaviors (e.g., kind and cultivating; Eagly and Karau, 2002).

#### Micro-Level

At this level, factors such as gender differences in career attitudes, choices, needs, motivation, and self-evaluation comes into play. Usually female employees must give more time to family, home, and care giving activities (Bianchi and Milkie, 2010), due to which they are not able to give due importance and time to their careers. It is also one of the reasons they lag in developing strong work-related networks that usually facilitates career progression. As compared to male employees, female personnel are inclined toward family and are “dual-centric” (Kossek and Lautsch, 2012), and have a stronger preference for work that provides flexibility for family and are heftier utilizers of flexibility practices (Kossek and Michel, 2011). These inclinations hold female’s progress as managers back. Usually administrators tend to promote work-centric personnel who are dedicated to becoming “ideal workers” and who prioritize paid work (Williams, 2000). Another interesting concept is that of queen bee syndrome (Staines et al., 1974). According to Derks et al. (2011) though it is occasionally established that women who attain powerful ranks are inspired to advance the career prospects of other women and become their role models, a number of studies have also demonstrated that powerful women restrain instead of boost the prospects of their female peers (e.g., Staines et al., 1974; Ellemers et al., 2004).

Nevertheless, again, it is vital to mention here the interplay of macro-level factors that shape the attitudes of women. In recent research, Derks et al. (2016) proposed that instead of being a foundation of gender disparity, the queen bee syndrome is itself an outcome of the gender bias that female personnel encounter at jobs. Women tend to internalize the customs and traditions of the culture they are part of. They realize from childhood that they do not enjoy similar rights as males and will behave in accordance with their gendered “social role” (Syed et al., 2018). Therefore, such behavior is not generally a feminine reaction but a portion of an overall self-group distancing response that is also observable in other marginalized groups. Additionally, the chance of a women being hired or promoted to an extent largely depends on family standing and socio-economic class, thus highlighting the crossroad of gender and status (Acker, 1999; Holvino, 2010). Status is a significant aspect of disparity and an unjust distribution of authority amongst women (Holvino, 2010).

In Pakistan, often “sifarish” (Islam, 2004) plays a vital part in employment and promotions. It denotes the course through which distinct ends are attained, frequently by means of personal contacts through people in influential positions, obtained from family relations or good friendships. Undeniably, “sifarish” potentially acts significantly in the career progression of female personnel since they require the endorsement and support of a male family member. A similar concept of wasta exists in Arab societies (Tlaiss and Kauser, 2011). In absence of sifarish, Pakistani women find it difficult to apply for jobs, get a promotion or training opportunities, as men usually control important networks with access to influential resources, contacts, and information. It is again vital to note that the connection of gender and socio-economic status is entrenched in meso-organizational and broader macro-national structures. At the meso-organizational level, gender and socio-economic interactions and stratification are formed into organizational structures, procedures and customs of doing work, creating and recreating disparity and privilege. On the national level, communal structures, views and ways of engaging construct and recreate disparity along the axes of status and gender (Holvino, 2010).

##### Social role theory at micro-level

Gender socialization processes, in line with the social role theory (Eagly and Steffen, 1986) inculcate gender roles throughout childhood (Lippa, 2005), that are strengthened at mature ages by the processes of expectancy confirmation (Powell and Greenhaus, 2010). People adopt the anticipated social roles and normalize their activities on the basis of gender-stereotypic beliefs (Wood and Eagly, 2009). Adopted gender stereotypes result in self-directed prejudice in women’s self-appraisal and they prefer to prioritize family and care-giving activities (Hakim, 2000), while men are expected to give preference to their careers. This might result in different labor market outcomes due to the internalization of gender roles at this micro-level. The literature review advocates a need to understand women’s career related issues in Pakistan holistically, using a multilevel analysis.

## Methodology

### Research Approach

It is important to select a suitable research approach and paradigm in accordance with research questions and context (Hair, 2010). Considering our research questions, an interpretive qualitative method was utilized (Saunders et al., 2012) for exploring the intricate features of Pakistani women’s lived experiences in work life. Such an approach is suitable for attaining a detailed understanding of particular contexts and settings (Cresswell, 2003). This exploratory study “gave voice” to the female experience and tried to enlighten their connotations and understandings (Creswell, 2013). In contrast to quantitative designs, qualitative methods offer profound insights into the assumptions and procedures underlying a phenomenon (Bryman, 2004), and permits a comprehensive investigation of the various contextual issues that might influence it (Bryman et al., 1996).

### Data Collection and Sampling Procedure

Carrying out research with women participants in conservative and patriarchal societies is a challenging task. Considering the difficulties in obtaining samples through conventional techniques, convenience, and snowball techniques were utilized (Berg et al., 2004). The intent behind such sampling was not to “establish a random or representative sample but rather to identify those who have information about the process” (Hornby and Symon, 1994, p. 169). A snowball method was utilized so that participants suggest other potential respondents (Atkinson and Flint, 2001). To avoid the risk of sample bias due to snowballing, where initial respondents may refer individuals whom they know very well with likely very similar traits and characteristics (Biernacki and Waldorf, 1981), five distinct snowball sequences were initiated, separately beginning from a diverse networking source, including initial respondents from the banking, telecommunications, healthcare, education, and food industries.

With the help of phone calls and emails, the potential respondents were updated regarding the research purpose. Before data collection started, all the participants were assured that this study will conform to suitable standards of data retention, confidentiality and ethics. All the participants provided their informed written permission for inclusion in the study before the commencement of data collection. To ensure the anonymity of participants, no identifying information was included in the manuscript. Letters (A, B, C…) were utilized in place of their names. The disclosed ages of the participants lie within the range of +3 and -3 of their real ages.

### Interviews

Our qualitative approach espoused data collection through interviews (Creswell, 2013). Twenty-seven in-depth semi-structured interviews were organized in line with specific protocols (see Appendix), adopted from Abalkhail (2017) and updated according to the requirements of the current study, though the interviewer was receptive to uncover other themes and matters touched on by the interviewees. Sometimes probing questions were deemed necessary to steer the interviews toward the basic aim of the study. Interviews were conducted face-to-face, on the phone, WhatsApp and Skype, each lasting from 40 to 75 min. Most of them were through video calls, to capture the non-verbal cues that have been contended to be imperative in producing rich qualitative data, particularly when investigating delicate topics like gender disparity (Sturges and Hanrahan, 2004). Eight interviews were conducted in English, while rest were conducted in Urdu and translated to English using a parallel translation by a couple of Urdu–English speakers. To warrant precision of the translation procedure, the final records were checked by a bilingual researcher. To reassure openness, interviews were not recorded, though detailed notes were taken throughout. To minimize data loss, two female assistants took thorough notes along with the interviewer.

The concept of reflexivity (Alvesson and Sköldberg, 2009) is central in guaranteeing the precision of qualitative data. The act of reflection permits a careful contemplation of the asymmetrical interface between interviewer and respondent and the means by which this interface might be compromised by presuppositions resulting from demographics or more elusive cues (Syed et al., 2018). An understanding of misperceptions through reflexivity allows the researcher to plan particular questions that assist, update and illuminate his/her interpretation of the outcomes (Roller and Lavrakas, 2015). The current research did not hold any such issues regarding misperception or misunderstanding of answers or cultural feelings since the interviewer, assistants, and respondents were of the same gender and socio-religious and cultural identity.

In order to add rigor, precision, and credibility several techniques were used including triangulation (Patton, 1999), confirmability (Shenton, 2004), and member checks (Mertens, 2014). To be more specific, investigator triangulation was utilized, where the involvement of two or more researchers was used (in the form of one interviewer and two assistants for data collection) in order to provide multiple observations and conclusions. Such a triangulation technique can fetch both confirmation of the conclusions and diverse perspectives, adding breadth to the phenomenon of interest (Carter et al., 2014). Member checks included rechecking with the respondents at the end of interviews if the researcher noted and summarized what had been stated correctly and inquired if the notes precisely reflected the respondent’s position. The draft of the final findings especially containing the quotes of respondents were shared and verified to ensure the credibility of the findings. All eight respondents interviewed in English were member checked whereas half of the Urdu speaking respondents verified the notes. Confirmability was also done using the same technique.

Despite the relatively moderate sample size, data collection was ceased only when saturation in data was attained after 27 interviews and no new information was being obtained. Table 1 presents the demographic information of the respondents.

**Table 1:** Respondent demographics.

Respondent	Education	Sector	Experience (year)	Age (year)
Ms. A	MBA	Banking	04	26
Ms. B	M. Phil, M.Ed.	Education	11	32
Ms. C	MSc, B.Ed.	Education	14	34
Ms. D	MBA	Telecom	08	29
Ms. E	Master	Telecom	18	37
Ms. F	M. Phil	Education	20	39
Ms. G	MBA	Banking	18	37
Ms. H	Ph.D.	Education	16	40
Ms. I	M.Com	Banking	09	30
Ms. J	Bachelor	Fast Food	03	22
Ms. K	BBA	Banking	06	26
Ms. L	MBA	Education	06	28
Ms. M	MBA	Banking	21	43
Ms. N	M. Phil	Education	25	47
Ms. O	MSCS	Education	10	33
Ms. P	MSc	Education	15	37
Ms. Q	Master	MNC	24	49
Ms. R	MBA	MNC	04	23
Ms. S	MBA	Telecom	05	26
Ms. T	MBBS, FCPS	Health	07	27
Ms. U	MBA	Banking	26	48
Ms. V	Bachelor	Fast Food	02	22
Ms. W	MBA	Banking	04	25
Ms. X	M.Com	Banking	06	28
Ms. Y	MBA	Banking	26	48
Ms. Z	MBBS, MD	Health	11	32
Ms. AA	Master	MNC	04	27


### Data Analysis

Each female respondent denoted a unit of analysis and was studied as a case. Notes were made right after each interview to ensure that important information was not forgotten or lost. Following the steps suggested by Miles and Huberman (1994) and Braun and Clarke (2006) for qualitative analysis, a continuous comparative thematic analysis was performed. After reading transcriptions word by word numerous times (Miles and Huberman, 1994), a coding scheme was established to depict the pertinent thoughts concentrating on specifics about work challenges, barriers, facilitators, and outcomes of gender equality at workplace. The codes were regularly revised, utilizing the procedure of analytic induction. Based on these codes, the main themes were generated. These were then re-assessed to guarantee coherency and termed afterward ensuing a thematic framework of related ideas.

**FIGURE 1:**
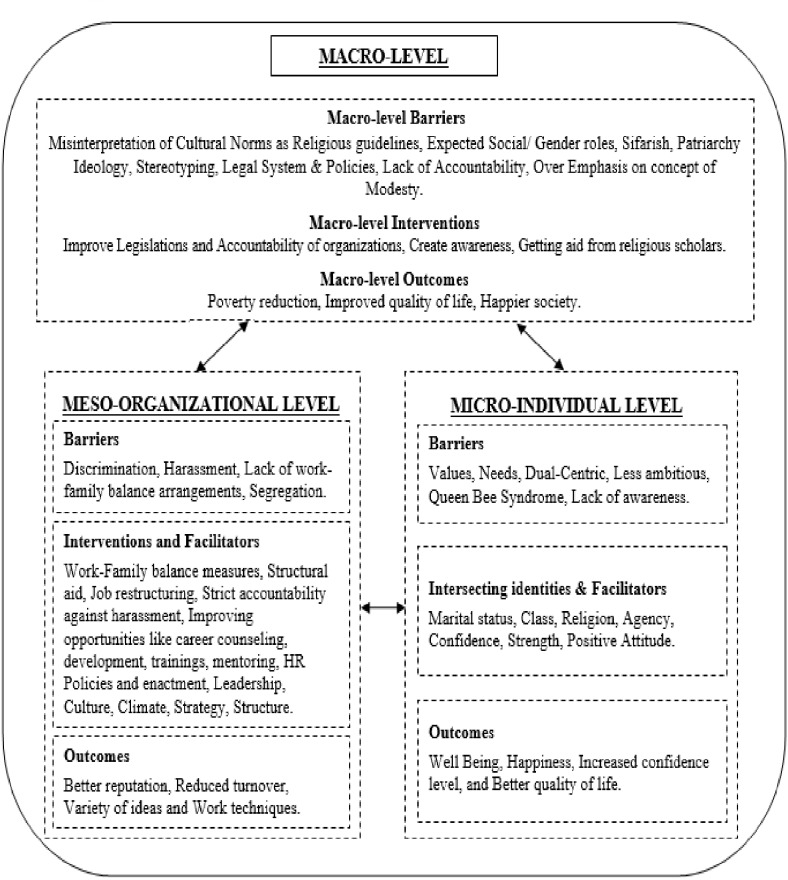
Multi-level interaction of factors related to workplace gender parity in Pakistan.

Though the original themes were verbalized in the voice of the respondents, the next stage led to a higher level of abstraction and stemmed through a review of the literature and lining up the codes with the current body of knowledge. Following this method, the basic ideas about workplace gender equality, processes and outcomes were obtained from the transcriptions in the form of major themes with the help of NVivo 12 plus which assisted in the thematic analysis and facilitated in mapping emerging associations among the themes (King, 2004). As a final step dimensions were gathered theoretically, and associations were built between the themes recognized in accordance with the research questions. The conclusions were then outlined around Syed and Özbilgin’s relational framework. The consequent outcomes were explained as themes and interconnections as depicted in Table 2 and visually displayed in the conceptual model (see Figure 1).

**Table 2:** Themes and subthemes identified in data on three levels.

Level	Themes	Sub-themes
Macro-level	Barriers	Islamic guidelines: cultural norms and traditions are misinterpreted as religious teachings.
		Patriarchal Ideologies: social norms favor male dominance and superiority while restricting women to homes and allows men to control various aspects of women’s lives. Sifarish: the social power of who you know and who you are related to.
	Interventions	Improve legislations and accountability of organizations. Getting aid from religious scholars regarding the interpretation of true meaning of Islamic teachings. Create awareness about the Islamic guidelines related to working women and their rights and duties.
	Outcomes	Poverty reduction: when women work and there are more dual earners, poverty is reduced. Improved quality of life: with better income, quality of life becomes better. Happier society: happier and confident women creates happier society and households.
Meso-level	Barriers	Discrimination: women employees being discriminated at workplace with respect to career start, development and success opportunities. Harassment: women employees being sexually and emotionally harassed my male colleagues. Lack of work-family balance arrangements; pushing mothers out of the workforce. Segregation at workplace where women are not considered appropriate for some jobs; men have strong networking ties that help them in career.
	Interventions	Work-family balance measures including flexible work, holidays, and daycares. Strict policies and accountability against harassment.
		Improving opportunities like career counseling, development, trainings, mentoring, structural aid, and job restructuring.
		Meritocratic HR policies and enactment, leadership, culture, climate, strategy, and structure of equality.
	Outcomes	Better reputation of Organizations as EEO and WFB providers therefore attracting larger pool of candidates in recruitment process. Reduces turnover and more satisfied workforce. Variety of ideas and work techniques that comes with diverse workforce.
Micro-level	Barriers	Values and Needs: women having a caring and sacrificing nature.
		Dual-centric career orientation: women being less ambitious and giving equal importance to home and family. Queen Bee syndrome: successful women not proving to be facilitating to women subordinates. Lack of awareness: women employees not aware of policies and procedures for their legal and emotional facilitation.
	I.D^∗^ and facilitators	Gender and marital status, class and religion: more than one identity intersecting within a woman thereby creating a web of career inhibiting factors. Agency and strength: women showed characteristic strength, agency, and resilience. Confidence and positive attitude: women seemed motivated to make positive change.
	Outcomes	Well-being and happiness. Increased confidence, self-esteem, and efficacy. Better quality of life.


*^∗^I.D, intersecting identities.*

## Findings

The emergent themes and subthemes, along with respondent’s relevant quotes highlighted several significant and interconnected issues from personal, organizational, and socio-national constraints; to the organizational interventions and mechanisms aimed at gender parity, and the outcomes thereof. It is vital to mention that these themes are intertwined because of the interaction between the connections at the micro, meso, and macro levels. Figure 1 presents an outline of the main themes and subthemes that transpired from the data. Though the findings are organized for each level, it is important to emphasize the interconnected character of the various aspects identified.

### Macro-Level

#### Barriers

The findings provide evidence about Pakistani women’s belief in national, socio-economic and the cultural context of Pakistan which plays a significant role in their career outcomes. Particularly, most of the women mentioned the misinterpretation of religious text with respect to women’s modesty as a major challenge. According to them, the cultural norms and traditions are misrepresented as the religious guidance that has resulted in negative stereotypes regarding women going out to earn money. Especially women’s expected gender roles as care givers in the society seriously harms them as careerists. One respondent mentioned: Islam is the religion of ease and does not impose any restrictions on women going out to earn money. If the couple in consultation with each other decides to be dual earners, it is fine in the eyes of Islamic teachings (Ms. G, Banking). Another working woman addressed this issue by saying that: even in this modern age, earlier misconceptions and stereotypes of male members of society exists toward career-oriented women. They think that women’s priority should only be house chores, care giving and serving men. Such men mingle religion with culture, traditions, and regulations which acts as a barrier for women to go and work outside the home. Patriarchal ideologies play a major part in this (Ms. L, Education).

Another interesting concept that emerged is of “Sifarish.” It is an Urdu language term that most closely relates to the notion of favoritism and nepotism in English. Almost all women participants expressed their disgust toward it and mentioned it as a widely prevalent plague in the society that affects everyone on merit in general, and women in specific. A female respondent from academia summarized it by saying: Sifarish is a major challenge for everyone who works hard and is on merit, but the problem is more resound for women as they mostly lack the quality and quantity of networks that men have in our society. Working women face this problem from entry level, to promotions, training, development opportunities, rewards, and benefits (Ms. B, Education). Additionally, Pakistani working women pointed out that though there have been reforms and additions in legislations that might ease working experiences of career women, the problems largely remain as is, because of a lack of accountability and implementation. In the words of a women manager: in Pakistan there have been additions of several legislative reforms from the government, but organizations just embrace them as formalities and such legislations have not significantly improved women’s career prospects due to deficiency in accountability (Ms. P, Education). All these factors have a trickle-down effect at meso and micro levels.

#### Interventions

Several important interventions were mentioned by the respondents that might improve women’s career prospects at this level. All the respondents put forward that the government must take appropriate steps not only in improving the legislations but also the process of accountability. Organizations and leaders must be held accountable for any gap in the legislative guidelines and their actions and practices. A young woman from the banking sector said: in our industry there have been many instances of harassment and discrimination. Banks adopt the equal opportunity and other laws just to fulfill the requirement from the government on paper. We know there are laws ensuring the rights of women, but we cannot follow them through as they are just formalities and the procedures are very time consuming and complex. The best solution would be accountability from government personnel; their regular visits and timely feedback might help solve the problem by implementing policies into practice (Ms. K, Banking). Additionally, more than half of the participants suggested that the government might accept help from religious scholars for a comprehensive comprehension of religious guidance in the matter of women and work and publicize it in media and social media to eliminate religious misperceptions and reduce stereotypes and gendered roles arising from traditions instead of religion. An associate professor shed her views by saying: a number of conservative men from our society have selected assorted Islamic teachings and utilize these to prove their supremacy over us (women), deciphering the Quranic guidelines in accordance with their male-controlled interests. It is the duty of the current government to look into the matter in these contemporary times and appoint moderately liberal Islamic scholars who can teach and spread the real meanings of Islamic guidelines and make ordinary men aware of what is actually required under the teachings of the Islamic umbrella (Ms. H, Education).

#### Outcomes

The most significant outcomes that all women stated would occur from career equality, leading to better female workforce participation, is poverty alleviation. One of the respondents working in a call center narrated that: I belong to a conservative lower-class background and was not allowed to work by my in-laws, though I am better educated than my husband. We faced a difficult time with five children as my spouse did not have a secure employment either. Later we, as a couple decided that I too will work and earn. Now we are happy and do not face much financial problems (Ms. E, Telecom). An expert of gender studies summarized it as: in Pakistan most of the population is below the poverty line or belong to the lower middle class or lower categories. Though there has been an increase in women’s education level, if it is also translated into their better workforce participation, it would work wonders in the reduction of poverty by a significant level, as dual earners have better prospects of an increased income household, which contributes to a better quality of living…. Other nations who have better female participation have also increased their GDP and reaped the benefits of increased productivity from a diverse workforce, due to gender inclusion (Ms. C, Education).

Another theme that appeared was the benefits associated to happiness. A respondent from the banking sector recalled that: earlier, when I did not work, our quality of life was poor. My husband did not earn enough. Because of the inferior financial situation, I used to remain agitated the whole day and beat up my kids. Since I have started working in the Bank, our quality of life has improved significantly, I stay happier now and my kids do not suffer anymore. I feel I can raise kids better now, though I face some shortage of energy and time to give to them (Ms. A, Banking). Another respondent from the banking sector shared similar views: I, from childhood, am a very independent kind of girl, full of confidence. I had been depressed immediately after marriage as I was not allowed to work for a couple of years due to modesty and honor issues from the in-laws. My health also started deteriorating. Once I started working again, I regained my health, confidence and happiness. My job gives me a feeling of competitiveness, independence and confidence. My job is one reason for my happiness and well-being (Ms. W, Banking).

### Meso-Level

#### Barriers

At the organizational level several important barriers affecting gender parity at the workplace emerged. Importantly many of these were linked with the socio-cultural, religious, and traditional norms about female employment that emerged from the macro level factors. The most commonly stated and significantly emphasized factor was gender discrimination at the workplace, leading to inadequate career prospects. According to a female respondent: there is a general discrimination that we face being women in getting a job, being promoted or getting training opportunities. When I entered my profession, I was the only female selected for the job out of eight selected candidates. Even in promotions and development opportunities, the HR managers prefer promoting men because they are considered to be the bread winners of the family. Many of my friends have not got any promotions even once in their career even though they have the required education, skills, and competence (Ms. O, Education). Another respondent stated: me and my husband started our jobs together with the same education, from the same University and in the same organization. My husband has been offered trainings many times and have been promoted twice in 5 years, whereas I am still at the same level and selected in training programs only once…. The reasons are embedded in our social norms where men are considered to be more competent and providers and protectors of females (Ms. Y, Banking).

Sexual harassment was another important issue that emerged from transcriptions. A respondent from the food industry stated: I face such acts of indecency on a daily basis. Being a waitress, I encounter a variety of customers. Many of them are young boys who make vulgar comments and inappropriate acts. Even sometimes I face the same issues from fellow employees (Ms. J, Fast Food Chain). A banking employee also added on the issue by saying that: my major concern is that we get promotion easily when we go out on dates and dinners with our boss. Otherwise hard work and merit are not considered enough for us to get promoted. Girls must seduce managers numerous times to achieve higher goals (Ms. W, Banking). The fact that such remarks come from women from a state that is reported to adhere to Islamic laws and ethics of modesty is an irony in itself suggesting the non-existence of authentic norms of inclusion and reverence for women.

Inadequacy or altogether lack of work-family policies and practices emerged as a major theme highlighted by three-quarter of married respondents. An assistant professor elaborated it by saying: juggling between work and family is a tough ask for women, since in our culture women have a specific gender role to take care of children, cook, clean, wash and manage the household, putting an extra burden of responsibilities on our shoulders. Lack of policies like working from home, giving online lectures, and no day care center, makes managing children, home, and work more difficult. I have three kids and it’s extremely tiring and exhausting for me to manage everything at once (Ms. N, Education).

Segregation is another important theme identified by the transcriptions, which is also influenced by macro level issues. Many jobs are considered to be appropriate only for men. One of the respondents working in a bank stated that: coming from a conservative family, the decision to work in a bank was a very difficult one, as everyone in family and circle of relatives criticized it. Though now more women have started working in banks, when I started my career, banks were segregated with males only. Even nowadays in our culture only a few professions are considered appropriate for women, like health care and teaching (Ms. X, Banking). Another subtheme related to segregation was networking. Respondents felt that there is gender related segregation especially in networking which is vital for careers in our cultural settings. One of the senior professors said: in our profession, knowledge exchange is vital for progressing in the career. In Pakistan usually, men have their own social circles which exclude women. Due to a lack of wide and diverse networks women usually lag in terms of publications and idea generation for research work. This hinders their promotion prospects (Ms. H, Education).

All these themes and sub themes illustrate interrelations, between macro and meso-level factors where factors at a national level trickle-down to organizations. The social norms and traditions widespread within the society are in turn found to be prevailing and practiced in organizations. Additionally, these factors also influence at the micro level, where individual choices, needs, and motivations are shaped by factors at a higher level.

#### Interventions

The respondents were asked about the factors they consider necessary for promoting gender equality within organizations and almost all respondents mentioned the role of the human resource (HR) department to be vital for the cause. Second most occurring subtheme was that of leadership. All the respondents put forward that without the commitment of senior and junior leadership such a goal is difficult to achieve. According to one lecturer: the HR department and leadership must work together in making appropriate policies, decisions, and the enactment thereof for gender inclusion…. Facilitating women in their careers requires building an organization’s strategy, structure, culture, and climate on the principals of gender parity and inclusion (Ms. F, Education). A young respondent working in an HR department proposed that: the HR department can play a crucial role in furthering the equality of women employees at work. It is of utmost importance that decisions regarding recruitment, selection, promotion, and rewards be taken strictly on merit instead of gender stereotypes. Career development opportunities must also be offered on merit instead on the assumption that since men are breadwinners, they deserve better opportunities at work…. The organizational leadership also plays a major part in promoting a culture, climate and strategies of gender equality in the work environment (Ms. S, Telecom).

More than two-third of the respondents had a view that presently not enough measures are being taken from organizations in Pakistan that can facilitate women in their careers. Though some of them mentioned that their organizations provided maternity leave, equal pay, and holiday when required due to family issues, half of them thought their organizations did not take any significant action for gender inclusion and equality. Amongst the important interventions that needs to be adopted by organizations, mentioned by the respondents, were flexible work arrangements, day care facilities, job restructuring, career counseling and mentoring, strict accountability on gender harassment, career development, and networking opportunities. A respondent summarized it by saying: the telecom industry here is not yet a viable one for married working women with children as compared to the education and healthcare industry. We need to have at least daycare centers and family related leaves in times of emergency. Flexible work arrangements, trainings, and mentoring would be an icing on the cake…. A culture of gender equality needs to be created especially in our industry [Ms. E, Telecom (mother of five kids)].

#### Outcomes

Numerous important outcomes for the organizations, from gender equality, were identified by respondents. A couple of benefits mentioned by three quarters of participants were those of organization’s reputation and reduced female turnover. An assistant professor mentioned that: my university offer better women friendly policies as compared to other universities in Pakistan. They have established a proper day care center beside the campus with proper arrangements. They provide extensive maternity benefits as well as off or half day off when required due to family reasons or a child’s ailment. …. My University also makes sure to avoid any harassment and discrimination in training and promotional opportunities for women. I am happy working here and don’t want to move from here, even if offered more pay from others …. In the last 5 years my university has gained a great reputation due to anti gender discriminatory policies and pro equality and inclusion. It is respected considerably because of this. A lot of women, especially married ones are willing to join it (Ms. C, Education).

Around half of the respondents believed that gender inclusion is helpful for organizations because of diverse techniques and ideas women bring into the workplace. A female manager working in a multinational company said: a number of times, in meetings, when we are stuck at some point, it sometimes happens that the female members suggest some brilliant out of the box ideas that surprises us but proves to be very useful…. Females have different ways of tackling tasks, sub ordinates and fellows. Most of the time, our employees are happier with female managers as compared to males (Ms. AA, MNC). Such comments provide evidence that organizations do and can reap numerous benefits from female participation in the workforce and if they provide a climate of equality and inclusion, they can significantly improve their own as well as women’s career states.

### Micro-Level

Micro level barriers and facilitators to career progression for women take the form of differing levels of motivation, ambition, values, needs, goals, agency, and resiliency. It is imperative to mention that the findings at this level, demonstrate an interconnection between respondent’s gender, religion, marital status, socio-economic class, marital status, and work experience.

#### Barriers

Interestingly, the transcriptions showed words like “giving,” “helping,” “caring,” “sacrificing,” “being considerate,” and “not hurting others” repeatedly. Such emotions, values, identities, and ideals play an imperative role in defining individual’s future choices, judgments, perceptions, and actions. In context of this study, these standards are found to be related to the career prospects of women. One respondent narrated an incidence: once we were working on an important project. Such projects help us in our promotion. I worked very hard on it and did 90% of the work. But my male colleague took all the credit for it in front of the boss. I ignored it as I didn’t want to hurt him by complaining. Also, he was a poor guy, so I sacrificed my credit for him (Ms. Q, MNC) …. mostly my female colleagues care for others at work and want to help…. Males on the other hand are more competitive and think more from mind then heart. Maybe it is because in our society, they are under pressure to run the income of the house (Ms. R, MNC).

Additionally, the findings showed women to be less ambitious and that they preferred to spend extra time with family, children and in care giving. More than half of respondents mentioned a personal need and interest in spending more time in family care, house responsibilities and almost all considered careers as secondary. One of the respondents summarized it as: I recall, in my childhood, my parents always suggested to me to learn house chores. They said it would be the only thing that would be helpful for me after marriage. They used to give advice to my brother to study and work hard on his career only, though I was a better student and used to get better grades than my brother. We have seen from our early years how the focus of our mother’s was on children, and father’s on income. So, it has been engraved in our minds to consider life this way (Ms. T, Health). It may be worth asserting here that the conception of care is associated with the historical social role of women especially in Islamic states and as a result is an adequate example of the interplay of various levels for understanding the difference in perceptions, ambition, values, and actions between genders.

Queen Bee syndrome was also an emergent and surprising theme from the findings. Half of the respondents believed that when women reach supervisory and leadership roles, instead of acting as mentors, role models and facilitators for female sub-ordinates they become harsh on them comparatively. Some of the respondents belonging to senior positions were enquired about it and one of them responded by saying: I faced a lot of gender bias on my way up and had to work so much harder to prove myself in this man’s world. It won’t be fair if now I give easy access to women just because of their gender. They must be willing to work hard as well (Ms. U, Banking). It is easy to infer from this statement that queen bee conduct is a reaction to the prejudice and social identity threat that Pakistani women face, though more research and evidence is needed to conclude this.

#### Intersecting Identities and Facilitators

It was observed that single females were normally more susceptible to gender harassment. A waitress working in a fast food chain related an experience of her fellow colleague by saying that: we face such acts of harassment on a daily basis, sometimes from customers, sometimes from colleagues…… A very close friend of mine faced an immense incident of harassment at work. She has not only left this job but now her family does not let her work anywhere (Ms. J, Fast Food). Another single respondent mentioned that: I left my last job because of harassment issues. Despite several complaints to management, they failed to take any significant action. After 1 year of mental torture I left that job…… occurrences of harassment are present here (at current job) as well but comparatively fewer (Ms. G, Banking). For married women, barriers as illustrated by their statements in the earlier section, took the form of conflict between domestic, social and work responsibilities and burden. Second, the notes unfolded intersections of gender and socio- economical class. The wealthier and influential women used sifarish and networks to get jobs and promotions. Women’s experiences of gender parity differed with respect to personal situations. For instance, one respondent gave a comparison with her friend: me and my best friend passed from the same university. I was among the top three students, but she got a handsome job within 3 months of passing, whereas I had to wait for over a year to get this job. Now after 4 years she has already been promoted to the next level; while I am still at the same entry level position…... She comes from an influential and wealthy background, her father being a bureaucrat. She has a lot of strong sifarish to back her career (Ms. I, Banking).

Finally, individual strength and agency emerged as repeating themes. More than seventy five percent of participants perceived themselves to be confident, bearing a positive attitude and self-efficacy. A woman in a senior management position shared that: it is very tough for women to commence a career in this country. Sifarish and bribing are more useful here as compared to formal qualifications and work experience. In such a situation, I have reached at this position only because of my untiring efforts, hard work, skills and a positive attitude toward career…. even sacrificing my family life occasionally (Ms. K, Banking). Another respondent described how she exerted strength and independence in accumulating valued experience and also being financially independent: I work majorly for three important reasons, first, it gives me a sense of achievement, second, it makes me feel independent financially and it boosts my self-confidence. To be honest, I am unwilling to be a financial burden on anyone, especially men. I choose to work very hard and struggle a bounty for that (Mr. Z, Health). The self-assurance and esteem mirrored in “I am unwilling to be a financial burden” demonstrates that Pakistani women cherish being financially independent and have the drive and determination to contest the status quo of gender disparity. It is imperative to mention here that most participants accredited the overall generally protecting character of Pakistani culture toward women backed up by their experience of given preferential treatment in bank lines, payment counters at grocery stores, bus queues and all women busses and canteens.

Interestingly the work experience of the respondents had a positive influence on their career encounters. Most of our interviewees with better work experience and who had been rewarded with pay and promotions were found to be more ambitious than the rest. Additionally, the more work experience they had, the better they stated was the quality and quantity of their networks which can further their advancement opportunities. The incidences of harassment also declined as the work experience increased. This might be attributed to age or marital status as well. Regrettably, our analysis also showed that more experienced and successful women indulged in behaviors like the queen bee syndrome, though such a conclusion requires further research.

#### Outcomes

Most of the outcomes at this level are related to the personal well-being of the respondents. As evident from the quotes in earlier sections, it emerged from the notes that the respondents mostly believed that having a gender inclusive work environment facilitates their work experiences and career progression which results in happiness, an increased confidence level, and a better quality of life. A response from a doctor practicing in Lahore city reflects these outcomes: I have always been very energetic and wanted to serve people as a doctor especially the poor ones from early childhood. Working as a medical doctor not only gives me a sense of independence and accomplishment but also heart felt happiness not only because of earning well and better life quality but also by being helpful to the needy… I work in the evening in a private clinic and provide free or cheap consultation to poor patients. It gives me a feeling of success and satisfaction in this world as well as the hereafter (Ms. Z, Health).

## Discussion

The findings of this study evidently demonstrate the interconnection of macro-, meso-, and micro-level factors in explaining career related experiences of Pakistani women and outcomes of an inclusive work environment and policies. Undoubtedly the analysis proved to be challenging due to the complexity of intertwining factors at the three levels of analysis.

The central religion followed in Pakistan is Islam, which plays a substantial role in shaping the socio-cultural state. While Islam accentuates the notion of gender parity, the patriarchal understanding of it concerning the female’s place in the community appears to be lagging (Karam and Afiouni, 2014). The patriarchal interpretations of Islamic guidelines emerge from concept of qiwama, which deals with the specifics of family life (spouses). Such an interpretation is in line with the notion of gender role segregation arising from the social role theory (Eagly and Steffen, 1986). This idea has been reflected by some as, the one who is employed and makes money will be in charge of the other (Abalkhail, 2017). It is commonly construed by some Muslim scholars as connotation that God made males superior to females (Mernissi, 2011). Consequently, males are given control of the female’s matters, both in the domestic and social life. The current study’s findings reveal that the understandings of qiwama, and dominance over women prevails at the workplace just like in households. However, this is against the Islamic guidelines which teaches a husband to bear similar household responsibilities as that of the wife, as evident from real life examples and practices of the prophet Muhammad (PBUH) as a husband. Additionally, it was reflected by the interviewees that family income is used to validate males instead of females for selection and promotion since men are believed to be the breadwinners of family, even if female employees have similar or even superior education, skills, training, expertise; and even if, in some instances, females are the breadwinners of the family. Accordingly, in Pakistan, women’s recruitment, selection, rewards, and promotions are connected to gendered social roles within the broader community. This finding parallels several other studies (Eagly and Carli, 2007; Acker, 2009; Abalkhail, 2017).

The current study also revealed that the majority of respondents were highly educated, yet their career experiences remained unsatisfying. The notion of the ‘leaky pipeline’ is familiar in literature, along with the ideas of ‘glass ceiling’ and ‘sticky floor,’ which explains the declining percentage of females progressing in the educational and career ladder (Winchester and Browning, 2015). The ‘pipeline’ loses more females as they continue to under-represent in various professions and positions (Antelo, 2015). For instance, in Pakistan, many female doctors drop out of the profession when they start a family (BBC News, 2015). The results of our study also point toward the phenomenon of the ‘bursting pipeline’ (Kassem, 2012; Karam and Afiouni, 2014), where women despite having the necessary education, knowledge, skills, and expertise find it exceedingly difficult to secure, in addition to continuing employment. This is attributable to the intersection of identity with the socio-economic class and macro level factor of Sifarish and social roles, prevailing in the society at large. The fact, that approximately 10,000 applications are received for admission in medical colleges for a mere 100 places, reflects the tough competition and shows that only the highly competitive students get admission and pass as doctors in Pakistan. The Pakistan Medical and Dental Council (PMDC), states that above 70% of medical students are females, still government statistics imply that the majority of these brilliant female undergraduate doctors do not essentially go on to practice the profession. Merely 23% of registered doctors are female (BBC News, 2015). It would be an interesting future research avenue to study the experience of female doctors and medical students and analyze the causes of such occurrences in detail as well as holistically.

However, our research also depicts stories of strength, confidence and determination, illuminating how several women tackled the social and organizational pressures by unparalleled devotion to education, skills and career, signifying individuality. Though, it must be recognized here that displays of such strength is easier for women belonging to a relatively higher socio-economic class. Undeniably, the junction of gender, marriage, religion, and class along the notion of Sifarish are strongly interrelated. The higher a person’s status, the greater is the probability of him/her accomplishing their goals through Sifarish. A similar concept of Wasta prevails in comparable patriarchal societies (Cunningham and Sarayrah, 1993; Al-Asfour et al., 2017; Syed et al., 2018).

Masculine supremacy, as mentioned earlier, is a continuing challenge in Pakistan. Therefore, the lack of support from the father or husband sometimes translates to career impediments for women of Pakistan. Segregation adds further barriers at all three levels, restricting career access and development opportunities for women. Here, not merely socially acceptable jobs are considered enough, like teaching and healthcare, but family honor and female modesty are similarly imperative. Mernissi (2011) claims that gender segregation relates to some Qur’anic interpretations that act as a reinforcer of masculine supremacy in major Islamic nations. The macro factors consequently affect arrangements and practices in organizations that influence gender parity. Acker (2009) puts forward that professions and careers might be gender segregated from the inside, yet she identifies that the degree and pattern of segregation differs between organizations. The results of this study also underline the impediments faced within the workplace in the form of discrimination and work overload due to diverging burdens of the job, household and family. Harassment by managers, peers and customers and the portraying of women as a female rather than a colleague also represents a major barrier toward career success. Furthermore, overall lack of daycare facilities and other strategies like flexible or part-time jobs were found, which are similar to earlier studies conducted in patriarchal societies (Al-Asfour et al., 2017; Syed et al., 2018). As our findings show a positive influence of women’s work experience on their career outlooks, if their job situation can be improved and they do not leave the pipeline in the most important years of their careers, it might play a catalytic role on furthering their prospects in paid work.

Though, some of the earlier studies (Eagly and Carli, 2007; Alimo-Metcalfe, 2010; Karam and Afiouni, 2014) have recognized that female’s career impediments are because of macro and meso factors, yet knowledge regarding the institutional factors (religion, and social norms related to patriarchy) along with the micro level factors and their intersection in Pakistani women is an original contribution of this study. This study not only explores the impediments, but also, through the eyes of women, what steps are being taken and what further steps need to be taken to enhance their employment experience. Furthermore, the study also highlights the outcome and benefits associated with gender inclusion at all three levels that are mainly related to the well-being of respondents, organizations, and society at large. Importantly, unlike some of the earlier studies (e.g., Abalkhail, 2017; Alhejji et al., 2018) that demonstrate a trickledown effect of impeding factors from the macro to meso-level, this study also contributes by showing that such influence can also work bottom–up. Women might not wholly affect the macro- national and meso organizational level features, it is on the micro-individual level that they convey and practice strength and independence for advancing gender parity.

Importantly, the findings of our study have endorsed the tenets of social role theory. At the macro-level, the quotes of our respondents (e.g., Ms. L, Ms. H, and Ms. W) demonstrate that in the Pakistani socio-cultural and religious environment, specific gender roles prevail, binding women to serve men, look after children and take care of household and not to indulge in paid work due to conservative traditions and its wrongful portrayal of as a religious requirement. At the meso-level, the effects of macro-level factors trickle down and define the extent to which management practices are gendered in execution in various Pakistani organizations; as also evidenced by the quotes from our respondents (e.g., Ms. O, Ms. S, Ms. Y, Ms. N, and Ms. X). In line with the social role theory, since men are given the role of breadwinners in Pakistan, and because women assume the role of care givers, men get a privileged position at jobs resulting in discriminatory practices at the meso-level. Additionally, at the micro-level, since such gender roles are inculcated in the minds from early childhood, as pointed out by Ms. T and Ms. R; they result in a female mind set as either being only care-givers or as dual-centric, which puts them at a disadvantaged position in their careers. Thereby, our findings approve the propositions of the social role theory at all three levels in the context of Pakistan.

## Conclusion

The research tried to illuminate the multilevel impediments, interventions, and outcomes related to gender parity with respect to the career experiences of Pakistani women. Based on 27 semi-structured in-depth interviews, we utilized a relational viewpoint for improved comprehension and analysis of complicated and interlinked factors of gender parity at a national, organizational, and individual level. Women workforce participation in formal employment is not on par with their educational attainment in Pakistan. The current study displays that an intricate net of interconnected factors harmfully influences female labor force participation. The interviewees explained how cultural traditions, customs, and religious beliefs influence their career prospects. The narratives of Pakistani women employees have seldom been represented in previous studies. Hence, the outlooks explored here can prove to be a stepping stone for academics and experts operating in gender related fields, Human Resource Management (HRM), and human resource development (HRD) in Pakistan.

### Implications for Practice

The present research has several valuable implications for Pakistani policy makers, organizations, HR personnel, researchers, and various stakeholders interested in facilitating women’s career prospects. First, without changing the patriarchal interpretations of Islamic guidelines and social role structure, only producing a bulk of highly educated women is least expected to drive social change. Islam is the religion that “gave women their basic rights centuries before the West did” (Khalaf, 2017). Islam confronted female infanticide in early Arab societies, encourages women to learn and work (Khalaf, 2017) and considers them equal to men with respect to Islamic rights and duties (Al-Hibri, 2000). Over the years, the faint boundary parting religion from norms and customs has been distorted, and the traditions are frequently misidentified as religious principles. Therefore, the notion of Islam being a confining influence for women is not utterly a correct one. Fundamentalist’s elucidations of verses from the Quran, along with long-standing patriarchal norms, principally inherited from the Indo-Pak sub-continent, lies at the heart of the conundrum of gender inequality in Pakistan; which is in sharp contrast to the teachings of the Holy prophet Muhammad (Peace be upon Him) and Quran who emphasized the quest for knowledge as the inherent duty of both genders (Al-Qur’an, (39:9); Al-Qur’an, (96:1-5); Ibn Maja in Al-Sunan, (1:18)) no. 224).

Parallel to earlier researchers (Özbilgin, 2006; Hennekam et al., 2017), the present study proposes that multilevel support and cultural transformations are vital to accomplish gender parity at the root rather than at a superficial level. For this, the regulatory authorities of Pakistan, need to address structural as well as cultural impediments concerning female workforce participation by enforcing regulatory and legislative reforms, and their complete accountability thereof. Religious thoughts and ancestral traditions about female workforce participation dominant in the community must be reconsidered with the help of religious scholars, to pave the way for positive change. In organizations, the HR department needs to concentrate on producing a work environment without gender bias, where promotion and development opportunities are based on merit rather than gender. The provision of work family benefit initiatives should also be ensured by the HR department (Kemp and Madsen, 2014).

For researchers, the study demonstrates various similarities and dissimilarities in contrast to western based women’s career related studies and provide avenues for further research. HRD practitioners should consider the particularities of the Pakistani context when creating a career expansion model for women. Such implications are well-timed and looked-for as Pakistan concentrates on evolving at various socio-economic, and political frontiers.

At the macro-level, female labor participation is directly and irrefutably associated with enormous gains (Loko and Diouf, 2009) including economic development like an increased GDP (Aguirre et al., 2012), increased labor productivity (Scott-Jackson et al., 2010) and reducing poverty in developing nations (Elder and Smith, 2010). At the meso-level, exercising gender parity might allow these organizations to advance more quickly as compared to others that do not adopt such practices (World Economic Forum, 2014). It enables the firms to extract maximum payback from prevailing human resources, with more advancement opportunities (Barsh and Yee, 2012). Several studies (Krishnan and Park, 2005; Smith et al., 2006; Francoeur et al., 2008) have shown positive results of gender diversity. Moreover, gender-diverse boards can enhance corporate governance by providing a wider range of perspectives (Organization for Economic Cooperation and Development [OECD], 2012). Consequently, the firms that do not invite, indorse and advance women, harm their enduring success. For those who do, the returns from gender diversity are obvious.

The implications of gender parity are wider than mere monetary goals. Women coexisting as half of the worldwide population, deserve equivalent access to well-being, schooling, impact, earning power, and political representation. Humankind’s shared growth and prosperity is contingent upon this fact (World Economic Forum, 2014). Furthermore, a successful career and enhanced professional growth enhances a person’s self esteem which subsequently leads to happiness (Baumeister et al., 2003). Happier women would have better capability to foster children into improved and efficacious people and promote a constructive domestic environment. Such reforms would potentially also result in women with greater negotiating power within the private and public sphere, also allowing them to serve as second-income earners; in turn creating grassroots support from families for a women’s career.

### Theoretical Implications

The research contributes significantly to the existing body of knowledge, by demonstrating a strong support for Syed and Özbilgin’s (2009) relational framework and social role theory (Eagly and Steffen, 1986) in the Pakistani context. We show how various factors at the three levels are interconnected to form a web-like structure. Several scholars (Zanoni et al., 2010; Schwabenland and Tomlinson, 2015) have invited contextual research and have proposed that diversity management and gender parity becomes a dynamic intricate construct (Hennekam et al., 2017) when context is considered. Our study also confirms this assertion in line with the social role theory in context of career related experiences of Pakistani women. We augment the multi-level framework by including Pakistani contextual understandings of gender roles and demonstrate that limiting academic and executive considerations to merely the meso-level might not be enough, as imparity is entrenched in an intricate arrangement of supremacy relations and practices (Ahonen et al., 2014). We illustrate that it is at the micro-individual level that young women are contesting these traditional customs and bringing about incremental changes. Though some dimensions are out of their direct reach, the respondents did show a positive attitude to eventually shift the impact on meso- and macro-levels.

Furthermore, we bring to light that the dimensions of the micro-level, meso-level and macro-levels not only operate top–down, but that change can also work bottom–up. Intersections of several aspects also emerged as a major finding. At the micro-level, the study underlines the internal heterogeneity of Pakistani women and reflects the intersection of gender with socio-economic status and family identity. Whereas gender studies usually look at one feature (i.e., gender), our findings bring a package of factors into consideration (e.g., gender, status, religion, marriage) when studying gender equality in Pakistan.

### Limitations and Future Research Directions

Though the current research contributes significantly to the existing literature, it has limitations, particularly regarding the sampling method and size. Yet, because data was collected in patriarchal and religious settings of Pakistan, accessing female respondents who are willing to openly talk about sensitive issues like gender bias, sexual harassment, and exploitation is not an easy job. The sample size limitation is supported by the logic that an exploratory phenomenological study’s power rests in choosing information-rich cases that can offer abundant information (Patton, 2002). Future researchers might augment this study by including the opinion and interpretations of other stakeholders like men, managers, religious scholars and public policy creators, in a similar multi-level study. Additionally, keeping in view, a dearth of studies related to the grave issues of Sifarish and corruption prevailing in Pakistan, we call on future researchers to investigate them thoroughly in general and in particular to women employees.

We also join Hodges (2017) to invite future researchers to study lived experiences of women entrepreneurs who are running personal businesses in Pakistan, focusing on the facilitators and impediments and determining if it is a practical way forward for other Pakistani women. It would also be interesting to carry out a cross-country study and utilizing mixed methodology research designs to comprehend women’s work-related prospects. Lastly, in line with the call from Syed et al. (2018) we recommend future researchers to study other probable intersections that might result in a better, more nuanced comprehension of gender disparity at the workplace.

## Ethics Statement

All participants of this study provided their informed written consent for inclusion in the study before data collection. The study was carried out in accordance with the Declaration of Helsinki. The researchers ensured that the proposal, procedures, and the protocols adopted in the study complied with the international suitable standards of data retention, confidentiality, and ethics in how the data would be treated. However, an ethics approval was not deemed necessary as per our institutional guidelines and national laws and regulations since nothing unethical has been carried out in our study. We just conducted in-depth interviews with the respondents, made notes and were exempt from further ethics board approval as this research did not involve human clinical trials, lab experiments, or animal experiments. Researchers maintained full confidentiality and anonymity of respondents throughout the manuscript. All participation was voluntary.

## Author Contributions

AS made a substantial contribution to the conception and design of the study, contributed in the acquisition, collection, and the organization of data, wrote the first draft of manuscript, made and designed the figure and tables. MI made a contribution in the analysis and interpretation of the data, aided in drafting the findings section and helped in working on NVivo. The majority of the contributions was made by AS during the revision stage. Both the authors read and approved the submitted version.

## Conflict of Interest Statement

The authors declare that the research was conducted in the absence of any commercial or financial relationships that could be construed as a potential conflict of interest.

## Appendix (Interview Protocols)

Following interview protocols, adopted from Abalkhail (2017) and updated in accordance with the aims and context of the study, were followed, after having expert opinion from academics and scholars:

(1) Which factors do you think impact women’s workplace equality (or inequality) and career progression at national/ societal level?
  (a) What initiatives can be taken to overcome them?
(2) Which barriers do you think impact women’s workplace equality and career progression within the organization/ workplace?
(3) Which personal weaknesses and/or strengths do you think impact women’s workplace equality and career progression?
(4) Which mechanisms in organizations do you think are vital for improving women’s career progression?
(5) What steps do you think organizations can take to improve career related opportunities for women?
  (a) And what steps are they already taking?
(6) What benefits would an organization get from such initiatives for improved gender diversity?
(7) What will be the benefits of such initiatives at national level?
(8) What steps do you think women can take to improve their career related opportunities?
(9) What will be the benefits of such steps for women personally?
(10) Why are there organizations where gender diversity is not achieved regardless of the efforts, they put to promote gender diversity?
